# Räumliche Assoziation zwischen Luftschadstoffen und Erkrankungen des Atemwegs- sowie des Herz-Kreislauf-Systems in Hamburg

**DOI:** 10.1007/s00103-025-04122-5

**Published:** 2025-08-27

**Authors:** Sandra Hischke, Simone Kühn, Volker Matthias, Martin Ramacher, Katharina Schulze, Jobst Augustin

**Affiliations:** 1 Institut für Versorgungsforschung in der Dermatologie und bei Pflegeberufen (IVDP), Universitätsklinikum Hamburg-Eppendorf (UKE), Martinistraße 52, 20251 Hamburg, Deutschland; 2 Zentrum für Umweltneurowissenschaften, Max-Planck-Institut für Bildungsforschung, Berlin, Deutschland; 3 Klinik und Poliklinik für Psychiatrie und Psychotherapie, Universitätsklinikum Hamburg-Eppendorf (UKE), Hamburg, Deutschland; 4 Institut für Umweltchemie des Küstenraumes, Helmholtz Zentrum Hereon, Geesthacht, Deutschland

**Keywords:** Luftqualität, Atemwege, Herz-Kreislauf, Raum, Stadt, Air quality, Respiratory, Cardiovascular, Space, City

## Abstract

**Hintergrund:**

Luftschadstoffe können Atemwegs- und Herz-Kreislauf-Erkrankungen begünstigen, dies haben zahlreiche Studien bereits nachgewiesen. In dieser Studie wurden räumliche Unterschiede der Luftschadstoffbelastung in Hamburg sowie deren Assoziationen mit Atemwegs- und Herz-Kreislauf-System-Erkrankungen untersucht.

**Methoden:**

In die ökologische Querschnittstudie wurden 9787 Teilnehmende der Hamburg City Health Study (HCHS) im Alter von 45–74 Jahren eingeschlossen, die zwischen April 2016 und November 2018 an der Basisuntersuchung teilnahmen und seit mindestens 10 Jahren am aktuellen Wohnort leben. Zur Ermittlung der Konzentrationen von O_3_, NO, NO_2_, PM_10_ und PM_2,5_ wurden Modelldaten des Helmholtz-Zentrums Hereon (EPISODE-CiTYCHEM) für das Jahr 2018 genutzt. Neben deskriptiven Auswertungen und kartografischen Darstellungen der Luftschadstoffbelastung und adjustierter Prävalenzen wurden räumlich logistische Regressionsmodelle verwendet.

**Ergebnisse:**

Es zeigen sich insbesondere erhöhte Feinstaubbelastungen im Zentrum von Hamburg, wo sich sowohl große Verkehrsachsen als auch der Hafen befinden. Zudem besteht ein positiver Zusammenhang zwischen der Feinstaubkonzentration und Erkrankungen des Atemwegs- sowie des Herz-Kreislauf-Systems.

**Diskussion:**

Die Ergebnisse decken sich mit vorangegangenen Studien. Die Wirkmechanismen bedürfen weitergehenden Untersuchungen, um insbesondere gezielte Maßnahmen zur Senkung des Erkrankungsrisikos ableiten zu können.

## Abstract (en)

**Background:**

Numerous studies have already shown that air pollution can contribute to respiratory and cardiovascular diseases. This study investigated spatial differences in air pollution in Hamburg and their associations with respiratory and cardiovascular diseases.

**Methods:**

This ecological cross-sectional study included 9787 participants in the Hamburg City Health Study (HCHS) aged 45–74 who took part in the baseline examination between April 2016 and November 2018 and had lived at their current place of residence for at least 10 years. Model data from the Helmholtz Centre Hereon (EPISODE-CiTYCHEM) for 2018 were used to determine the concentrations of O_3_, NO, NO_2_, PM_10_ and PM_2.5_. In addition to descriptive analyses and cartographic representations of air pollution exposure and adjusted prevalence rates, spatial logistic regression models were used.

**Results:**

Increased particulate matter pollution is particularly evident in the centre of Hamburg, where both major traffic arteries and the port are located. In addition, there is a positive association between particulate matter concentration and diseases of the respiratory and cardiovascular systems.

**Discussion:**

The results are consistent with previous studies. The mechanisms of action require further research in order to be able to derive specific measures to reduce the risk of disease.

## Einleitung

An der Entstehung respiratorischer und kardiovaskulärer Erkrankungen sind neben genetischen Faktoren auch Umweltfaktoren wie Feinstaub oder Lärm beteiligt [1]. In urbanen Räumen ist die Belastung durch Luftschadstoffe hervorzuheben, da diese über die Atemwege sowohl akute als auch chronische Erkrankungen verursachen können [2]. Von besonderer Bedeutung sind dabei kleinere Partikel [3]. Zahlreiche Studien haben Langzeiteffekte von Feinstaub (Particulate Matter, PM) verschiedener Größenklassen mit einem aerodynamischen Durchmesser ≤ 2,5 µm (einschließlich Ultrafeinstaub ≤ 0,1 µm) sowie ≤ 10 µm (PM_2,5_, PM_10_), Stickoxiden (NO, NO_2_, NO_x_) und Ozon (O_3_) auf die Gesundheit untersucht [4–6].

In einer in 29 europäischen Städten durchgeführten Studie wurde ein linearer Zusammenhang zwischen einem Anstieg der PM_10_-Konzentration um 10 µg/m^3^ und einem Anstieg der Mortalität aufgrund von Atemwegserkrankungen nachgewiesen [7]. Darüber hinaus fanden Cesaroni et al. [8] eine statistisch signifikante positive Assoziation zwischen langfristiger NO_2_- und PM_2,5_-Exposition und kardiovaskulärer, ischämischer Herzkrankheit und Lungenkrebssterblichkeit. Insbesondere Adam et al. [9] wiesen darauf hin, dass eine erhöhte Feinstaubbelastung am Wohnort zu einer Verminderung der Lungenfunktion führen kann. Die gleichen Effekte wurden auch bei NO_2_ beobachtet. XU et al. [10] fanden einen Zusammenhang zwischen der Zahl der Notfallaufnahmen aufgrund von chronisch obstruktiver Lungenerkrankung (COPD) und der Luftschadstoffkonzentration in Peking. Ein Anstieg der PM_2,5_-Konzentration um 10 µg/m^3^ war mit einem Anstieg der Notfallaufnahmen am selben Tag um 1,46 % verbunden. Wurden 3 weitere Tage berücksichtigt, stieg die Zahl der Einweisungen um 3,15 %.

Im Hinblick auf Herz-Kreislauf-Erkrankungen haben Hoffmann et al. [11] gezeigt, dass eine langfristige Belastung durch Verkehrsemissionen eine erhöhte Prävalenz von Durchblutungsstörungen des Herzmuskels begünstigt. Es gibt auch Hinweise darauf, dass Feinstaub nicht nur das Risiko für kardiovaskuläre Ereignisse wie Herzinfarkt oder plötzlichen Herztod bei bereits bestehender arteriosklerotischer Grunderkrankung erhöht, sondern sogar zur Entstehung und zum Fortschreiten von Arteriosklerose beiträgt [12]. Des Weiteren deutet eine wachsende Zahl epidemiologischer und experimenteller Studien darauf hin, dass Luftschadstoffe wie PM_2,5_ akut zu einem Anstieg des Blutdrucks beitragen können [13]. Speziell länger andauernde oder kumulative Luftschadstoffbelastungen mit NO_2_ scheinen hierbei von Bedeutung zu sein [14]. Für Myokardinfarkte konnten Mustafic et al. [15] zeigen, dass auch akut erhöhte Partikelkonzentrationen mit einem steigenden Risiko verbunden sind. So wurde für eine PM_10_-Erhöhung um 10 µg/m^3^ ein um 0,6 % erhöhtes Myokardinfarktrisiko festgestellt. Für NO_2_ wurde ein Anstieg des Herzinfarktrisikos um 1,1 % bei einem Anstieg um 10 µg/m^3^ festgestellt.

Die gesundheitlichen Auswirkungen der Exposition gegenüber Luftschadstoffen unterliegen jedoch einer hohen räumlichen Dynamik [16]. Dies gilt insbesondere für städtische Räume und den (räumlichen) Zusammenhang zwischen Luftschadstoffen und damit verbundenen Krankheiten, der bisher nicht vollständig verstanden ist. Die Auswirkungen variieren und sind abhängig von der geografischen Lage und dem urbanen Kontext. Insbesondere in Städten mit hoher Verkehrsdichte, industrieller Produktion und Hafenanlagen sind Emissionen von Luftschadstoffen wie Feinstaub und Stickoxiden stark ausgeprägt. In manchen Wohngebieten trägt die Holzverbrennung zur Wärmeerzeugung (z. B. Kaminöfen) zusätzlich zu einer hohen Feinstaubbelastung bei [17]. Das erhöht die Belastung der Bevölkerung durch Atemwegs- und Herz-Kreislauf-Erkrankungen. Viele Studien haben den Zusammenhang zwischen urbaner Luftverschmutzung und gesundheitlichen Auswirkungen bereits nachgewiesen, oftmals jedoch nicht innerstädtisch (nach Stadtteilen) differenziert.

Dieser Umstand betrifft auch die Stadt Hamburg, die mit fast 2 Mio. Einwohnerinnen und Einwohnern die zweitgrößte Stadt Deutschlands ist. Die Zunahme des maritimen Schiffsverkehrs durch die Globalisierung und des weltwirtschaftlichen Wachstums führt zu einer erhöhten Belastung durch Luftschadstoffe [18]. So beschreiben Sorte et al., dass weltweit die mittlere Konzentration der NO_2_-Belastung in Häfen zwischen 12 µg/m^3^ und 107 µg/m^3^ liegt und die PM_2,5_-Konzentration zwischen 2 µg/m^3^ und 62 µg/m^3^ [18]. Speziell für Hamburg lässt sich in Bezug auf den zentral gelegenen Hafen und die damit assoziierten Schiffsemissionen feststellen, dass bereits im Jahr 2012 der Anteil der durch Schiffsverkehr verursachten NO_2_-Emissionen bei 36 % und der PM_10_-Emissionen bei rund 8 % lag – im Vergleich zu anderen Emissionsgruppen wie Straßen- und Luftverkehr, Hausbrand und Industrie. Besonders Stadtteile im Westen Hamburgs sind stark betroffen, was unter anderem auf die Emissionen anliegender Schiffe an den Containerterminals zurückzuführen ist [19]. Hamburg stellt daher eine bedeutende Untersuchungsregion dar, um die gesundheitlichen Auswirkungen von Luftverschmutzung zu analysieren. Die Stadt ist einerseits geprägt durch Industrie, ein hohes Verkehrsaufkommen und den größten Flughafen Norddeutschlands, andererseits durch einen hohen Anteil an Grünflächen. Wie in vielen anderen Großstädten zeigt sich auch hier ein soziales Gefälle innerhalb der Bevölkerung [20]. Darüber hinaus ist Hamburg mit der „Hamburg City Health Study“ (HCHS) Heimat der größten lokalen Gesundheitsstudie weltweit, die eine umfassende Datengrundlage bietet [21].

Das Ziel dieser Studie ist es, die räumliche Assoziation zwischen ausgewählten Luftschadstoffen, wie Feinstaub, Ozon und Stickstoffoxiden, und respiratorischen bzw. kardiovaskulären Erkrankungen in Hamburg zu analysieren.

## Methoden

### Daten

Diese ökologische Querschnittstudie basiert auf den Daten der ersten 10.000 Teilnehmenden der Hamburg City Health Study (HCHS; [21]), einer bevölkerungsbasierten Kohortenstudie in Hamburg. Die Kohorte wurde aus der zufälligen Stichprobe (koordiniert durch das Einwohnermeldeamt) von Einwohnern im Alter von 45 bis 74 Jahren im Zeitraum von April 2016 bis November 2018 (Basisuntersuchung) gebildet. Berücksichtigt wurden bei der Analyse nur Probandinnen und Probanden mit einer Wohndauer von mindestens 10 Jahren am derzeitigen Wohnort, um sich möglichen Langzeiteffekten des Wohnumfelds anzunähern. Die Wohndauer (in Jahren) wurde per Selbstangabe erfasst. Als Endpunkte wurden folgende respiratorische und kardiovaskuläre Erkrankungen einbezogen: Asthma und chronische Bronchitis/chronisch obstruktive Lungenerkrankung (COPD), Hypertonie, koronare Herzkrankheit, Herzinfarkt und Schlaganfall. Die Angaben hierzu erfolgten per Selbstbericht anhand eines Fragebogens im Rahmen der Basisuntersuchung. Die Endpunkte wurden jeweils in die Diagnosegruppen aggregiert. Sofern eine der Erkrankungen aus der Diagnosegruppe auftrat, wurde eine Probandin oder ein Proband als „betroffen“ angesehen. Patientinnen und Patienten, die keine dieser Erkrankungen in der jeweiligen Diagnosegruppe aufwiesen, galten als gesund.

Um die Konzentrationen der Luftschadstoffe O_3_, NO, NO_2_, PM_10_ und PM_2,5_ zu berücksichtigen, wurden Modelldaten des Helmholtz-Zentrums Hereon, Abteilung für Chemietransportmodellierung, verwendet. Für die Modellierung wurde das Chemietransportmodell EPISODE-CiTYCHEM (Karl et al. [22, 23]) verwendet, welches angetrieben von regionalen Konzentrationen der europäischen Reanalyse (ein Verfahren, bei dem Wettermodelle und historische Daten genutzt werden, um langfristige Wetterdatensätze zu erstellen) von Luftschadstoffen des Copernicus Atmospheric Monitoring Service (CAMS; [24]) stündliche Konzentrationen der berücksichtigten Schadstoffe für das Jahr 2018 simuliert hat. Neben der Berücksichtigung von regionalen Luftschadstoffkonzentrationen durch CAMS werden in EPISODE-CiTYCHEM die Emissionen aus allen relevanten Quellgruppen (Industrie, Verkehr, Schifffahrt, Heizung etc.) für alle Schadstoffe berücksichtigt und deren chemische Umwandlung sowie deren Transport unter Berücksichtigung von stündlicher Meteorologie basierend auf physikalischen Prinzipien berechnet. Die simulierten Stundenwerte für das Jahr 2018 wurden mit Messungen des Hamburger Luftmessnetzes verglichen, um die Güte der Modellergebnisse zu evaluieren. Je nach Schadstoff kommen hier unterschiedliche Referenzmessverfahren an unterschiedlichen Messorten für die kontinuierlichen Messungen zum Einsatz. Für eine detaillierte Auflistung aller Verfahren und Stationen verweisen wir auf die Website des Hamburger Luftmessnetzes^1^. Die Simulation umfasste das gesamte Hamburger Stadtgebiet mit einer räumlichen Auflösung von 100 × 100 m für ein Gebiet von 50 × 50 km. Für die Verwendung in dieser Studie wurden aus den stündlichen Konzentrationsfeldern Jahresmittelwerte gebildet. Da aufgrund des Studiendesigns keine längsschnittliche Beobachtung möglich war, wurde für die Analyse der Feinstaubbelastung der Jahresmittelwert gewählt. Diese Entscheidung basiert auf der Annahme, dass der Jahresmittelwert eine gute Annäherung an die langfristige Belastung darstellt und somit geeignete Informationen für die Untersuchung gesundheitlicher Auswirkungen bietet.

Die modellierten Daten wurden im Studienzentrum des HCHS mit den HCHS-Daten verschnitten, sodass für alle Studienteilnehmenden ein jährlicher Durchschnittswert für 2018 pro Luftschadstoffvariable vorhanden war.

Als Kovariaten wurden in dieser Studie neben dem Alter zum Zeitpunkt der Basisuntersuchung und Geschlecht der Person auch der Rauchstatus (Rauchen: Nie/Früher/Ja) sowie der sozioökonomische Status (SES) einbezogen. Er umfasst einen Index-Score-Wert zwischen 3 und 21: Je höher der Wert, desto höher ist der sozioökonomische Status. Eine ausführliche Beschreibung ist in Klimesch et al. zu finden [25].

Des Weiteren war für jeden Studienteilnehmenden der Stadtteilcluster nach Erhardt et al. [20] des Wohnorts bekannt. Hamburg hat insgesamt 104 Stadtteile, wobei einige Stadtteile zu einem Stadtteilcluster (Mindesteinwohnerzahl *n* = 10.000) zusammengefasst wurden, um stabilere Schätzungen aufgrund der Einwohnerzahl sowie alters- und geschlechtsgruppenspezifischen Auswertungen machen zu können [20]. Die Teilnehmenden der 10.000er-HCHS-Kohorte verteilen sich auf insgesamt 68 Stadtteilcluster.

### Methodik

Zunächst wurden die Daten deskriptiv dargestellt. Kontinuierliche Variablen wurden als Mittelwert mit Standardabweichung und kategoriale Variablen als absolute Zahlen mit Prozentsätzen dargestellt. Weiterhin wurden die Daten zur kartografischen Veranschaulichung auf Stadtteilclusterebene aggregiert. Die geschätzten Prävalenzen der Erkrankungen wurden zuvor für Alter, Geschlecht, Rauchstatus und SES adjustiert und auf Grundlage eines gegebenen Werts der adjustierenden Variablen (Alter = 62,3 Jahre, Geschlecht = weiblich, Rauchstatus = rauchend, SES = 12,6) dargestellt. Das Alter und der SES sind die Mittelwerte der untersuchten Kohorte.

Um die Assoziation zwischen der Luftschadstoffbelastung und den respiratorischen sowie kardiovaskulären Erkrankungen zu untersuchen, wurden räumlich logistische Regressionsmodelle gerechnet. Grundlage bildet ein generalisiertes gemischtes Modell mit einem räumlichen autoregressiven Prozess, um die räumliche Abhängigkeit der Daten zu berücksichtigen. Es wurde eine binäre Nachbarschaftsmatrix gewählt, um die jeweiligen direkten Nachbarn der Stadtteilcluster zu berücksichtigen. Die Beschreibung einer derartigen Matrix ist in Wolf et al. [26] zu finden.

Jede Endpunktgruppe (kardiovaskuläre und respiratorische Erkrankungen) wurde separat analysiert. Für jedes Modell wurde jeweils eine Luftvariable als erklärende Variable auf den Krankheitsstatus (Erkrankt: Ja/Nein) verwendet und für Alter, das biologische Geschlecht, den Rauchstatus und den SES adjustiert. Die Inferenz basiert auf einer Bayes-Statistik unter Verwendung von Markov-Chain-Monte-Carlo-Simulationen (MCMC), die für 5000 Stichproben mit einem Burn-in von 1000 Iterationen durchgeführt wurden [27]. Das hier verwendete Modell bietet eine flexible und robuste Möglichkeit, räumliche Abhängigkeiten in den Daten zu modellieren, da es räumlich autokorrelierte und unabhängige Zufallseffekte miteinander kombiniert. Weitere Details sind in Lee et al. [28] zu finden.

Für jedes Modell wurde weiterführend die Prävalenz-Odds-Ratio (POR) mit zugehörigem 95 %-Glaubwürdigkeitsintervall berechnet. Ein 95 %-Glaubwürdigkeitsintervall, das den Wert 1 nicht enthält, wurde als statistisch signifikant angesehen. Die Datenanalysen sind mit R, Version 4.0 [29], und dem R‑Paket CARBayes [28] durchgeführt worden. Die kartografische Darstellung wurde mit ArcGIS Pro [30] visualisiert.

## Ergebnisse

### Deskriptiv

#### Kohorte.

Von der ursprünglichen 10.000er-Kohorte sind *N* = 9787 Probandinnen und Probanden aufgrund der Wohndauer von mindestens 10 Jahren in die Analyse eingeschlossen worden. Hiervon waren 48,4 % weiblich (5,5 % keine Angabe; Tab. 1). Das Durchschnittsalter lag bei 62,3 Jahren (SD 8,4). Es gaben 34,0 % an, dass sie noch nie geraucht haben, wohingegen 41,5 % früher geraucht haben und 18,5 % bis heute rauchen (6 % keine Angabe). Der durchschnittliche SES lag bei einem Index-Score-Wert von 12,6 (SD 3,5). Aus der respiratorischen Erkrankungskohorte haben 1139 von 8561 Teilnehmenden mindestens eine der respiratorischen Erkrankungen angegeben. Aus der kardiovaskulären Erkrankungskohorte haben 5879 von 8812 Teilnehmenden mindestens eine der kardiovaskulären Erkrankungen angegeben. Die Gruppe der kardiovaskulär erkrankten Personen war im Durchschnitt 6 Jahre älter als jene ohne diese Erkrankungen. Respiratorisch Erkrankte waren häufiger weiblich, kardiovaskulär Erkrankte häufiger männlich. Aktuell Rauchende waren häufiger bei den respiratorisch erkrankten Teilnehmenden zu finden. Der SES war in der Gruppe ohne die jeweilige Erkrankung höher. Der jährliche rohe Durchschnittswert der Luftschadstoffkonzentration lag bei den respiratorisch Erkrankten für alle Luftwerte bis auf O_3_ über dem Wert der Nichterkrankten. Bei den kardiovaskulären Erkrankungen war ausschließlich der mittlere rohe O_3_-Wert höher als bei der gesunden Vergleichsgruppe (Tab. 1).

**Tab. 1:** Beschreibung der Studienpopulation. Von der 10.000er-Kohorte wurden *N* = 9787 Teilnehmende eingeschlossen, die eine Wohndauer am aktuellen Wohnort von mindestens 10 Jahren haben. *Datenquelle*: Hamburg City Health Study [21]

*Studienpopulation*						
		Gesamt	Keine respiratorische Erkrankung	Respiratorische Erkrankung	Keine kardiovaskuläre Erkrankung	Kardiovaskuläre Erkrankung
*N*		9787	8561		8812	
			7422	1139	2933	5879
**Soziodemografie**						
*Alter*	In Jahren, M (SD)	62,30 (8,4)	62,18 (8,4)	62,38 (8,3)	58,70 (7,8)	64,38 (8,0)
*Geschlecht*	Weiblich, *n* (%)	4741 (48,4)	3718 (50,1)	651 (57,2)	1806 (61,6)	2715 (46,2)
	Männlich, *n* (%)	4506 (46,0)	3704 (49,9)	488 (42,8)	1127 (38,4)	3164 (53,8)
*SES*	M (SD)	12,62 (3,5)	12,76 (3,5)	12,18 (3,5)	13,04 (3,4)	12,40 (3,5)
**Risikofaktoren**						
*Rauchen*	Noch nie, *n* (%)	3326 (34,0)	2719 (36,6)	372 (32,6)	1078 (36,8)	2101 (35,7)
	Früher, *n* (%)	4062 (41,5)	3290 (44,3)	485 (42,6)	1203 (41,0)	2695 (45,8)
	Ja, *n* (%)	1812 (18,5)	1375 (18,5)	276 (24,2)	648 (22,1)	1049 (17,8)
**Luftschadstoffe**						
*O*_*3*_	In µg/m^3^, M (SD)	54,82 (10,0)	54,86 (10,0)	54,53 (10,0)	54,47 (9,9)	55,00 (10,0)
*PM*_*10*_		13,43 (1,2)	13,42 (1,2)	13,48 (1,1)	13,47 (1,2)	13,40 (1,2)
*PM*_*2,5*_		9,46 (0,8)	9,46 (0,8)	9,49 (0,7)	9,49 (0,8)	9,45 (0,8)
*NO*_*2*_		12,92 (4,8)	12,88 (4,8)	13,11 (4,6)	13,10 (4,8)	12,81 (4,8)
*NO*		5,16 (6,7)	5,16 (7,0)	5,24 (5,4)	5,22 (6,0)	5,12 (7,0)

*SES* sozioökonomischer Status, *M* Mittelwert, *SD* Standardabweichung, *O*_*3*_ Ozon, *PM*_*10*_ Particulate Matter mit aerodynamischem Diameter ≤ 10 µm, *PM*_*2,5*_ Particulate Matter mit aerodynamischem Diameter ≤ 2,5, *NO*_*2*_ Stickstoffdioxid, *NO* Stickstoffoxid

#### Luftschadstoffkonzentrationen 2018.

**Abb. 1:**
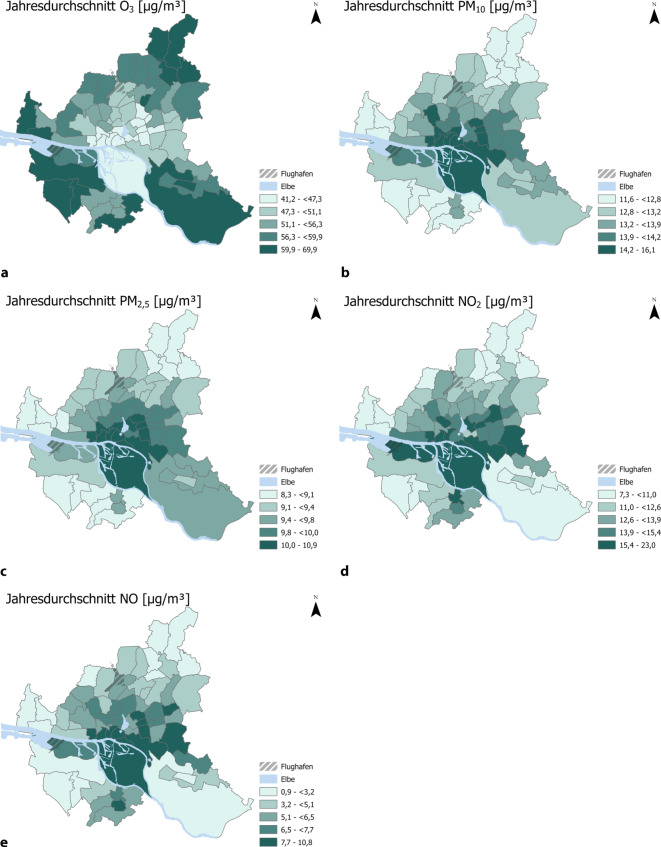
Jährliche Durchschnittswerte der Luftschadstoffe: **a** O3, **b** PM10, **c** PM2,5, **d** NO2 und **e** NO auf Ebene der Stadtteilcluster. *Datenquelle*: Modellierte Daten des Helmholtz-Zentrums Hereon (EPISODE-CiTYCHEM [22, 23], 2018)

Abb. 1 zeigt pro Stadtteilcluster den Jahresdurchschnitt der Luftschadstoffdaten von a) O_3_, b) PM_10_, c) PM_2,5_, d) NO_2_ und e) NO. Für die O_3_-Werte sind hohe jährliche Durchschnittswerte insbesondere am Stadtrand zu beobachten. Einer der zentralen Stadtteilcluster hat mit 41,2 µg/m^3^ die niedrigste jährliche durchschnittliche O_3_-Konzentration in der Luft. Im Nordosten von Hamburg liegt mit 69,9 µg/m^3^ die höchste durchschnittliche O_3_-Konzentration vor. PM_10_ und PM_2,5_ haben hohe Durchschnittswerte im Zentrum von Hamburg. Der niedrigste Jahresdurchschnitt eines Stadtteilclusters liegt mit 11,6 µg/m^3^ im Südwesten Hamburgs, der höchste liegt mit 16,1 µg/m^3^ PM_10_-Konzentration in Hafennähe. Auch bei den PM_2,5_-Werten liegt mit einem durchschnittlichen Jahreswert von 10,9 µg/m^3^ der höchste Wert in Hafennähe und der niedrigste mit 8,3 µg/m^3^ im Südwesten von Hamburg. Für NO_2_ und NO sind insbesondere in Hamburg-Mitte hohe Durchschnittswerte zu beobachten. Die Werte für NO_2_ reichen dabei von 7,3 µg/m^3^ (Südwesten) bis 23,0 µg/m^3^ (Hafennähe), für NO von 0,9 µg/m^3^ (Norden) bis 10,8 µg/m^3^ (Zentrum, Hafennähe).

#### Prävalenzen.

**Abb. 2:**
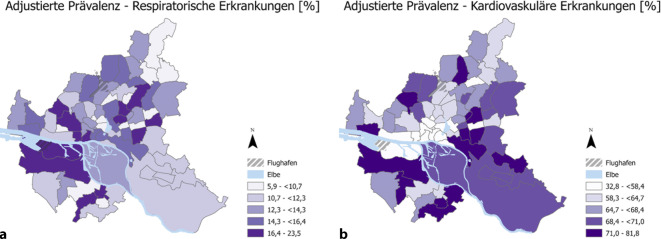
Räumliche Verteilung der nach Alter, Geschlecht, Rauchstatus und SES adjustierten durchschnittlichen Prävalenzen **a** respiratorischer und **b** kardiovaskulärer Erkrankungen auf Stadtteilclusterebene. *Datenquelle*: Hamburg City Health Study [21]

In Abb. 2 sind die nach Alter, Geschlecht, Rauchstatus und SES adjustierten auf Stadtteilcluster gemittelten Prävalenzen a) respiratorischer und b) kardiovaskulärer Erkrankungen abgebildet. Alles in allem ist bei beiden Erkrankungen ein eher diffuses Bild über die Stadtteilcluster zu beobachten, wobei insgesamt bei den respiratorischen Erkrankungen eine höhere Rate in Nähe des Stadtkerns, bei den kardiovaskulären Erkrankungen eine höhere Rate im Süden und Südosten von Hamburg vorhanden ist.

Die adjustierte Prävalenz respiratorischer Erkrankungen ist mit 23,5 % im Osten Hamburgs am höchsten. Mit 5,9 % besitzt der Nordosten die niedrigste adjustierte Prävalenz. Bei den kardiovaskulären Erkrankungen sehen wir insgesamt eine höhere adjustierte Prävalenz. Die höchste ist im Norden mit 81,8 % beobachtbar, die niedrigste mit 32,8 % in Hafennähe. Die rohen Prävalenzraten zu den respiratorischen und kardiovaskulären Erkrankungen der HCHS-Teilnehmenden in den Stadtteilclustern unterscheiden sich nur geringfügig von den adjustierten Prävalenzen und sind hier nicht abgebildet.

### Räumliche Regressionsanalysen

Die adjustierten räumlich logistischen Regressionsanalysen ergeben eine statistisch signifikante positive Assoziation zwischen PM_10_ bzw. PM_2,5_ und einer respiratorischen bzw. kardiovaskulären Erkrankung. Demnach zeigt sich im Modell für eine respiratorische Erkrankung ein POR von 6,8 % pro 1 µg/m^3^ PM_10_-Anstieg und ein POR von 8,2 % pro 1 µg/m^3^ Anstieg von PM_2,5_. Für eine kardiovaskuläre Erkrankung zeigt sich ein POR von 4,9 % (pro 1 µg/m^3^ Anstieg von PM_10_) bzw. 6,1 % (pro 1 µg/m^3^ Anstieg von PM_2,5_). O_3_ weist einen statistisch signifikanten, allerdings geringen negativen Zusammenhang mit der Erkrankungswahrscheinlichkeit auf. NO und NO_2_ sind statistisch nicht signifikant (Tab. 2).

**Tab. 2:** Posterior-Prävalenz-Odds-Ratios (POR) mit zugehörigen 95 %-Glaubwürdigkeitsintervallen der räumlich logistischen Regressionsmodelle, adjustiert für Alter, Geschlecht, Rauchstatus und SES. *Datenquellen*: Hamburg City Health Study [21]; modellierte Daten des Helmholtz-Zentrums Hereon (EPISODE-CiTYCHEM [22, 23], 2018).

	POR [95 %-Glaubwürdigkeitsintervall]				
	O_3_	PM_10_	PM_2,5_	NO_2_	NO
*Respiratorisch*	0,991* [0,985 0,997]	1,068* [1,029 1,103]	1,082* [1,008 1,147]	1,009 [0,996 1,020]	1,002 [0,992 1,010]
*Kardiovaskulär*	0,994* [0,991 0,997]	1,049* [1,028 1,068]	1,061* [1,027 1,092]	1,000 [0,993 1,006]	1,002 [0,998 1,006]

*Statistisch signifikant; *O3* Ozon, *PM*_*10*_ Particulate Matter mit aerodynamischem Diameter ≤ 10 µm, *PM*_*2,5*_ Particulate Matter mit aerodynamischem Diameter ≤ 2,5, *NO*_*2*_ Stickstoffdioxid, *NO* Stickstoffoxid

## Diskussion

Im Fokus dieser Studie stand die Frage, ob das lokale Auftreten von ausgewählten Luftschadstoffen in räumlicher Assoziation mit kardiorespiratorischen Erkrankungen steht. Die Ergebnisse am Beispiel Hamburgs zeigen, dass es innerstädtisch Unterschiede sowohl in der Luftschadstoffkonzentration als auch in der Prävalenz ausgewählter luftschadstoffassoziierter Erkrankungen gibt. Die Feinstaub- und Stickstoff(di)oxid-Belastung ist insbesondere in Stadtteilen, in denen der Hafen und große Straßenverkehrsachsen präsent sind, erhöht. Dies deckt sich mit Erkenntnissen aus früheren Studien von Sorte et al., die im globalen Kontext ebenfalls Häfen als eine substanzielle Schadstoffquelle identifizieren, beschreiben und berichten, dass dort häufig Grenzwerte überschritten werden [18, 31]. Besonders Stadtteile im Westen Hamburgs sind von einer erhöhten Stickstoffbelastung betroffen, was ebenfalls schon für das Jahr 2012 von Volker et al. nachgewiesen werden konnte. Dies ist unter anderem auf anliegende Schiffe an den Containerterminals zurückzuführen [19]. Hinzu kommt die durch den Straßenverkehr verursachte Schadstoffbelastung, die ebenfalls mit den Ergebnissen früherer Studien übereinstimmt (z. B. Zhang und Battermann [31]). Die Ozonbelastung ist am Stadtrand am höchsten. Diese Beobachtung deckt sich mit den Angaben des Umweltbundesamts, das für urbane Gebiete im Vergleich zu ländlichen Regionen niedrigere durchschnittliche Ozonkonzentrationen ausweist.^2^

Bei der Untersuchung, inwieweit die Luftschadstoffe mit den Erkrankungen zusammenhängen können, wurde die räumliche Abhängigkeit der Daten berücksichtigt und es zeigt sich in den adjustierten Modellen, dass sowohl PM_10_ als auch PM_2,5_ einen statistisch signifikanten positiven Zusammenhang mit einer respiratorischen und kardiovaskulären Erkrankung haben. Dies entspricht auch den Ergebnissen früherer Studien (z. B. Dominski et al., Rajagopalan et al., Rojas-Rueda; [1, 2, 32]).

O_3_ ist nach Adjustierung statistisch signifikant negativ mit den Erkrankungen assoziiert, NO und NO_2_ sind statistisch nicht signifikant. Da sich die Ozon- und Feinstaubkonzentrationen gegenläufig verhalten, lassen sich die in den Regressionsmodellen gefundenen negativen Zusammenhänge zwischen Ozon und Erkrankungswahrscheinlichkeit vermutlich dadurch erklären, dass in Gebieten mit hohem Verkehrsaufkommen die Ozonbelastung niedrig und die Feinstaubbelastung hoch ist. Dass hohe Ozonwerte eher im ländlichen Gebiet und nicht in Straßennähe gemessen werden, ist deutschlandweit beobachtet worden [33]. Der Zusammenhang lässt sich durch die NO-Titration erklären: In verkehrsreichen Gebieten entstehen hohe Konzentrationen von Stickstoffmonoxid (NO) durch Verbrennungsprozesse. Dieses NO reagiert mit Ozon (O_3_) zu Stickstoffdioxid (NO_2_), wodurch die Ozonkonzentration lokal reduziert wird. In ländlichen Gebieten mit weniger Verkehr fehlt diese titrierende Wirkung, sodass dort höhere Ozonwerte gemessen werden.

Diese Studie weist Limitationen auf. Wenngleich sich spezifische räumliche Assoziationen zwischen der Luftqualität und den berücksichtigten Diagnosen zeigen, können aufgrund des ökologischen Studiendesigns keine gesicherten kausalen Rückschlüsse gezogen werden. Dies betrifft beispielsweise die Luftbelastung in Hafennähe und das Auftreten von respiratorischen Erkrankungen. Darüber hinaus stellen die Daten zur Luftqualität jeweils Jahresmittelwerte bezogen auf den Wohnort der Studienteilnehmenden dar. Die tatsächliche Belastung einer jeden Person ist somit nicht im Detail gegeben, da die Luftzusammensetzung einer hohen räumlichen und zeitlichen Dynamik unterliegt [16]. Weitere Einflussgrößen wie Lebensstilfaktoren (z. B. Ernährung, Bewegung) oder das Freizeitverhalten konnten nicht berücksichtigt werden, sodass ein potenziell verzerrender Effekt nicht ausgeschlossen werden kann. Da gesundheitlich riskante Lebensstilfaktoren jedoch häufig mit einem niedrigen SES einhergehen [34], wurden diese Effekte indirekt berücksichtigt und einer Überadjustierung von Modellen vorgebeugt. Gleiches gilt für die Schadstoffexposition am Arbeitsplatz. Zudem beruhen die hier berücksichtigten Erkrankungen auf Selbstangaben und sind keine klinisch gesicherten Diagnosen. Entsprechend kann es auch hier zu Verzerrungen aufgrund eines Recall- oder Social-Desirability-Bias kommen.

Ferner unterliegt die HCHS auch einem Selektionsbias bezüglich des Alters, Wohnorts oder auch des Sozialstatus. Dies wurde bereits in Augustin et al. diskutiert [35], indem die ebenso hohen Prävalenzraten von Hypertonie innerhalb der HCHS beschrieben worden sind.

Trotz der vor allem methodisch bedingten Limitationen können aus der vorliegenden Studie sowohl wichtige Erkenntnisse als auch weitere Forschungsfragen abgeleitet werden. Künftige Studien sollten dabei beispielsweise kleinsträumig die Bedeutung des Hafens im Hinblick auf entstehende Emissionen und deren möglichen gesundheitlichen Auswirkungen auf die lokale Bevölkerung untersuchen. Bislang ist der Kenntnisstand dazu noch unzureichend. Darüber hinaus sollte auch die Frage der Umweltgerechtigkeit stärker in den Vordergrund gerückt werden, da sozial benachteiligte Gruppen, in diesem Fall auch Stadtteile, häufiger höheren gesundheitlichen Risiken durch Luftverschmutzung ausgesetzt sind.

## Fazit

Die hier gezeigten Ergebnisse auf kleinräumiger Ebene in Hamburg decken sich zum Teil mit bereits veröffentlichten Studien aus anderen Regionen. Es sind räumliche Assoziationen zwischen Luftschadstoffen und Erkrankungen der Atemwege sowie des Herz-Kreislauf-Systems identifizierbar. Der genaue Zusammenhang sowie die Wirkmechanismen bedürfen allerdings weiterer Untersuchungen. Da sich die Angaben der Erkrankungen auf Selbstangaben der Studienteilnehmenden stützen, sollte eine Untersuchung mit gesicherten ärztlichen Diagnosen angestrebt und zum Beispiel im Rahmen einer genesteten Kohortenstudie durchgeführt werden.

## Data Availability

Die im Rahmen der aktuellen Studie erstellten und/oder analysierten Datensätze sind aufgrund rechtlicher Beschränkungen nicht öffentlich zugänglich. Für eventuelle Rückfragen ist das HCHS-Studienzentrum (hchs@uke.de) zu kontaktieren.

## Footnotes

1 https://luft.hamburg.de (zugegriffen: 19.05.2025).

2 https://www.umweltbundesamt.de/daten/luft/ozon-belastung (zugegriffen: 14.05.2025).
